# Race modifies default mode connectivity in Alzheimer’s disease

**DOI:** 10.1186/s40035-020-0186-4

**Published:** 2020-02-19

**Authors:** Maria B. Misiura, J. Christina Howell, Junjie Wu, Deqiang Qiu, Monica W. Parker, Jessica A. Turner, William T. Hu

**Affiliations:** 1 Department of Psychology, Georgia State University, Atlanta, GA USA; 2 Departments of Neurology, Emory University, 615 Michael Street, Suite 505, Atlanta, GA 30322 USA; 3 Departments of Radiology, Emory University, Atlanta, GA USA

**Keywords:** Alzheimer’s disease, Cognitive impairment, Functional connectivity, Default mode network, Disparities

## Abstract

**Background:**

Older African Americans are more likely to develop Alzheimer’s disease (AD) than older Caucasians, and this difference cannot be readily explained by cerebrovascular and socioeconomic factors alone. We previously showed that mild cognitive impairment and AD dementia were associated with attenuated increases in the cerebrospinal fluid (CSF) levels of total and phosphorylated tau in African Americans compared to Caucasians, even though there was no difference in beta-amyloid 1–42 level between the two races.

**Methods:**

We extended our work by analyzing early functional magnetic resonance imaging (fMRI) biomarkers of the default mode network in older African Americans and Caucasians. We calculated connectivity between nodes of the regions belonging to the various default mode network subsystems and correlated these imaging biomarkers with non-imaging biomarkers implicated in AD (CSF amyloid, total tau, and cognitive performance).

**Results:**

We found that race modifies the relationship between functional connectivity of default mode network subsystems and cognitive performance, tau, and amyloid levels.

**Conclusion:**

These findings provide further support that race modifies the AD phenotypes downstream from cerebral amyloid deposition, and identifies key inter-subsystem connections for deep imaging and neuropathologic characterization.

## Introduction

It is not well understood why older African Americans are twice as likely to develop Alzheimer’s disease (AD) as older non-Hispanic Caucasian Americans (abbreviated as Caucasian hereafter) [1, 2]. While vascular disease [3–5] has been speculated to contribute to the disparities in AD risks, genome-wide association and clinical studies suggest race/ethnicity (hereafter referred to as race) also independently modifies the molecular pathways implicated in the development and manifestation of AD pathology. For example, the *APOE* ε4 allele confers lower AD risks for African Americans than Caucasians [6, 7], the *ABCA7* risk allele confers greater AD risks for African Americans than Caucasians [8], and AD is associated with less amnestic baseline performance and slower longitudinal decline in African Americans than Caucasians on neuropsychological analysis [9]. These cohort-level differences may reflect intrinsic biological differences between race, lower correlation between clinically-suspected and pathologically-confirmed AD (~ 75% accurate), recruitment bias in one or both races, or a combination of these factors [10, 11]. Data-driven strategies are therefore necessary to provide mechanistic correlates of observed race-associated differences to more clearly understand AD disparity.

One such approach is to use etiologic biomarkers associated with hallmark AD pathology to enhance the likelihood that those clinically suspected to have AD indeed have the pathology. We recently showed that in a group of older adults with mild cognitive impairment (MCI) or AD dementia, African Americans had lower cerebrospinal fluid (CSF) levels of tau-related biomarkers than Caucasians [12]. This is despite similar changes in CSF levels beta-amyloid 1–42 (Aβ42). We interpreted these findings as preliminary evidence for divergent biomarker trajectories and these differences have now been validated in one independent cohort in St. Louis as well as an independent younger cohort in Atlanta [13, 14].

Because we have not identified a difference in atrophy patterns on MRI between African Americans and Caucasians with AD, we hypothesized that resting-state functional MRI (rsfMRI) would be a more sensitive approach to identify the effect of race on AD-related neurological changes. We are particularly interested in resting state functional connectivity, as alterations in connectivity can be detected well before symptom onset [15] and track disease progression [16]. To explore brain changes associated with AD which may differ between races, we analyzed functional connectivity (hereafter referred to as connectivity) in the default mode network (DMN) using rsfMRI. The DMN is considered a potentially useful imaging biomarker for AD that is more widely available than amyloid PET [17–22].

In older adults, the DMN is broadly defined as correlated Blood Oxygen Level Dependent (BOLD) signal among the precuneus, posterior cingulate cortex (PCC), the inferior parietal lobule (IPL) and the ventromedial prefrontal cortex (vmPFC) [20, 23]. The DMN overlaps with anatomical sites vulnerable to amyloid deposition and atrophy in early AD [24], and reduced connectivity between DMN nodes (intra-network connectivity) mirrors the stage-wise tau deposition on PET imaging [25, 26] even before there is detectable atrophy [3]. The trajectory of AD functional connectivity changes is complex. The overwhelming majority of studies examining four DMN nodes reported reduced connectivity in AD (dementia) [27–31], with an exception reporting increased connectivity during early MCI [27]. However, few studies used etiologic biomarkers to distinguish between cognitive impairment due to AD, psychiatric illness, or cerebrovascular disease [32]. DMN hyperconnectivity has also been observed in asymptomatic *APOE* ε4 carriers when compared to non-carriers [33, 34], sometimes decades before symptom onset [35]. DMN connectivity may therefore have different relationships with AD risks (including risk genes), pathologic markers, clinical phenotypes, and disease stage, making inclusion of etiologic and clinical biomarkers in AD-related DMN analysis critical to ensure the consistency of findings.

As research on the DMN progresses, further fractionation of this complex network has revealed synchronous BOLD activity in regions outside traditional definitions of the DMN. Core subsystems [36] (dorsomedial, medial temporal lobe, and midline core) have been proposed to each contain key regions which work in tandem to support cognitive processes in learning and memory, retrieval of autobiographical information, self-referential processes [37], and social processing [38]. Dividing the DMN into its subcomponents has thus far provided more sensitive timelines for disease progression in AD and other neurological disorders [28]. Studies have shown that connectivity within the medial temporal lobe, rather than average DMN connectivity between the four core nodes, more consistently relates to cognitive impairment in AD [16, 30]; increases in connectivity within the anterior subsystems during early AD is more consistently identified in studies analyzing DMN subsystems [30, 34]; and memory impairment can be associated with decreased intra-subsystem connectivity within the medial temporal lobe [28] but increased connectivity between dorsomedial and midline core subsystems [39, 40].

The vast majority of studies analyze connectivity changes within diagnostic categories of normal cognition (NC), MCI, and AD dementia. Given that differences in cognitive impairment between NC and MCI and between MCI and AD dementia can sometimes be small, a continuous measure of cognition is preferred [41, 42] especially when it remains controversial whether current diagnostic algorithms are valid in African Americans (even with race-adjusted norms) [3, 43]. Thus, we also use a composite measure of cognitive performance derived from neuropsychological tests [12] to serve as a continuous, rather than categorical, measure of disease burden. We hypothesized that race modifies the relationship between connectivity and AD-related cognitive impairment, and between connectivity and two CSF AD biomarkers (Aβ42 [44] and t-Tau [45]). Furthermore, we specifically tested the generalizability of AD-associated connectivity changes between DMN nodes and between DMN subsystems to extend the AD biomarker phenotype in African Americans.

## Methods

### Participants

This study analyzed previously collected data from a study that recruited self-reported Non-Hispanic Caucasians and African Americans over the age of 65 across the diagnostic spectrum of Alzheimer’s disease dementia including individuals with normal cognition (NC), individuals with mild cognitive impairment (MCI), and individuals with Alzheimer’s Disease (AD) [12]. The study was approved by the Emory University Institutional Review Board. Each participant underwent a detailed interview for demographic information, self-reported race (Caucasians of Hispanic or Latino ethnicity were not included in this study), vascular risk factors (coronary artery disease, congestive heart failure, atrial fibrillation, hypertension, hyperlipidemia, diabetes, suspected transient ischemic attack), other medical comorbidities (e.g., cancer), and medications (e.g., use of angiotensin-converting enzyme inhibitors or angiotensin II receptor blockers). Each participant was then assigned a diagnosis according to consensus criteria including those for NC, MCI, and AD dementia (global Clinical Dementia Rating of 1 or 2.) Cognitively impaired subjects suspected of having a non-AD dementia (vascular, Lewy body, and frontotemporal dementia) were excluded. While our cohort was not age and gender matched specifically, we did not find significant differences in age or gender between races (Table 1). As previously reported, diabetes and hypertension were more common in African Americans than Caucasians, but African Americans had lower brain total white matter hyperintensity (WMH) volumes than Caucasians. Demographic data in Table 1 refer to individuals who passed MRI quality control standards (*n* = 137) as described below.

**Table 1:** Demographic information for the final cohort who passed imaging quality control process

	Overall (*N* = 137)		Normal cognition		MCI		AD	
	Caucasian (*n* = 72)	African American (*n* = 65)	Caucasian (*n* = 31)	African American (*n* = 27)	Caucasian (*n* = 25)	African American (*n* = 28)	Caucasian (*n* = 16)	African American (*n* = 10)
Age, years (SD)	70.90 (7.84)	69.20 (7.49)	71.65 (8.39)*	67.48 (6.17)*	71.52 (5.82)	70.07 (7.74)	68.50 (9.41)	71.4 (9.65)
Women (Men)	35 (37)	43 (29)	19 (12)	17 (10)	15 (10)	12 (16)	9 (7)	6 (4)
Years of Education (SD)	16.32 (2.85)	16.15 (2.85)	16.81 (2.69)	15.78 (2.66)	17.08 (2.58)	16.29 (2.81)	14.19 (1.94)	16.80 (3.55)
MMSE (SD)	26.32 (4.90)	26.91 (3.57)	28.84 (0.86)	28.00 (1.86)	27.64 (1.68)	26.67 (2.34)	21.37 (3.38)	20.22 (6.18)
Cognitive Z Scores (SD)	−0.78 (1.12)	−0.72 (0.99)	0.07 (0.51)	−0.11 (0.51)	− 0.88 (0.86)	− 0.67 (0.59)	−2.24 (0.66)	−2.36 (1.02)
White Matter Hyperintensity volume (mm^3^)	3984.40 (4110.10)	3886.03 (4964.93)	3306.81 (3337.32)	2440.93 (2576.15)	4018.66 (4080.03)	4313.70 (5330.60)	5243.68 (5333.14)	6730.21 (7607.48)
Having *ABCA7* risk allele, (%)	23.67*	43.10*	29.00	44.40	24.00	50.00	12.50	30.00
Have Diabetes, (%)	5.67**	33.80**	6.50**	33.33**	0*	28.60*	12.50*	50.00*
Have Hypertension (%)	45.83**	72.31**	45.16	62.96	56.00	78.57	31.25*	80.00*
CSF Aβ42, pg/ml (SD)	148.35 (95.02)	168.48 (128.86)	199.93 (132.30)	165.27 (89.8)	199.11 (141.02)	134.69 (90.99)	77.65 (47.39)	133.55 (140.71)
CSF t-Tau, pg/ml (SD)	72.84 (49.63)**	40.92 (20.79)**	51.68 (30.96)**	36.04 (11.63)**	63.99 (33.71)*	35.44 (13.67)*	108.48 (77.43)	75.8 (33.52)
CSF p-Tau_181_, pg/ml (SD)	17.58 (8.97)**	25.81 (12.47)**	23.47 (9.71)**	14.05 (5.07)**	25.01 (13.42)*	17.74 (7.21)*	33.53 (14.64)	24.54 (10.27)
CSF t-Tau/Aβ42	0.73 (0.91)*	0.24 (0.22)*	0.46 (0.74)*	0.15 (0.12)*	0.44 (0.42)*	0.22 (0.14)*	1.36 (1.16)	0.55 (0.41)

Note: T-tests were performed to compare races in the whole cohort and within each diagnostic category. *p < .05, **p < .001. MMSE and CogZ scores were significantly different between diagnostic categories such, NC having highest MMSE scores and AD having lowest (MMSE: NC vs MCI, *p* = .007, MCI vs AD, *p* < .0001, CogZ: NC vs MCI, *p* < .0001, MCI vs AD, *p* < .0001)

### Cognitive, genetic, and CSF biomarkers

Neuropsychological analysis was performed as previously described [12]. Briefly, each subject underwent a detailed neurologic examination and neuropsychological analysis. These included (1) memory (Consortium to Establish A Registry for Alzheimer’s Disease word list delayed recall, Brief Visual Memory Test–Revised [BVMT-R] delayed recall), (2) executive function (Trail Making Test B, reverse digit span [RD], Symbol Digit Substitution Test, and letter-guided fluency), (3) language (Boston Naming Test [60 items], category fluency), and (4) visuospatial function (Judgment of Line Orientation [JOLO], Rey-Osterrieth complex figure test). With the exception of BVMT-R, JOLO, and RD, subtest Z-scores were calculated according to published normative data, adjusting for age, sex, education, and race. Z-scores for these three subtests were calculated using the same norms in Caucasians, but calculated using Atlanta-based, cognitively normal African Americans because published norms generated mean Z-scores of > 2. Domain-specific Z-scores were calculated by averaging subtest Z-scores, and Z-scores for the four domains were averaged to generate composite cognitive Z-scores. Subjects with MCI and AD dementia had lower MMSE and cognitive Z-scores than subjects with NC (*p* < 0.01 for all comparisons, Table 1). In addition, each subject underwent standardized collection of blood (for *APOE* and *ABCA7* genotyping) and CSF without overnight fasting according to a modified Alzheimer’s Disease Neuroimaging Initiative (ADNI) protocol as previously described [46].

### MRI acquisition and preprocessing

Each subject underwent MRI scanning using a modified ADNI protocol on a 3 T scanner (Siemens AG) which included a T1-weighted 3D MPRAGE sequence (TR/TI/TE = 1620/950/3 msec, flip angle = 30^o^, matrix = 192 × 256 × 160, and voxel size = 0.98 × 0.98 × 1 mm^3^) and a 6 min eyes-open resting state functional MRI scan (TR/TE = 3000 ms/32 ms flip angle = 90^o^, field of view (FOV) = 200 × 200 mm^2^, acquisition matrix = 64 × 64, voxel size = 3.1 × 3.1 × 3.5 mm^3^, slice = 33, time point = 124) at the Emory Center for Systems Imaging. For rsfMRI, we used the DPABI v4.0.190305 toolbox to preprocess the image data [47] after discarding the first 10 volumes to allow the magnetization to approach a dynamic equilibrium, and to allow for more time for our participants to get comfortable inside the scanner [31, 48]. Individual echo-planar imaging (EPI) data were slice time corrected. Participants whose head motion exceeded 3.0 mm in translation or 3° in rotation were excluded. We further reduced the confound of head motion by higher-order regression based on Friston’s 24-parameter model [49], and the effect of physiological artifacts by covarying signals from CSF space and white matter [50]. EPI data were normalized to a study specific template generated using the DARTEL algorithm in DPABI that is better suited for populations with larger amounts of atrophy than standard normalization to the MNI template [51]. A spatial filter of 6 mm full width at half maximum Gaussian kernel was used. Subsequently, a band pass temporal filter (0.01–0.08 Hz) was applied to reduce the low-frequency drifts and high-frequency noise.

### MRI quality control

To be eligible for this analysis, participants must have had a T1 suitable for use in segmentation, as well as a usable resting state scan. To further eliminate confounds from head motion, we removed anyone whose mean framewise displacement (MWFD) was 3 mm and higher [52]. Among 145 subjects, 8 (5%) had rsfMRI that did not pass quality control and were excluded from DMN analysis. Table 1 displays demographic data only for individuals included in the MRI analysis (*n* = 137), and Table 2 shows demographic data for individuals not included in the analysis. Compared to those included the analysis, those excluded did not differ significantly in age, gender, diagnosis, or race. There was no significant difference in motion according to race or diagnosis.

**Table 2:** Demographic information of individuals excluded from analysis who did not pass QC

	Overall (*N* = 8)		Normal cognition		MCI	
	Caucasian (*n* = 5)	African American (*n* = 3)	Caucasian (*n* = 4)	African American (*n* = 2)	Caucasian (*n* = 1)	African American (*n* = 1)
Age, years (SD)	69.78 (5.83)	65.20 (10.25)	71.65 (8.39)	67.48 (6.17)	71.52	70.07
Years of Education (SD)	16.82 (2.85)	16.25 (2.63)	16.84 (2.69)	16.78 (2.66)	17.08	16.29
MMSE (SD)	27.82 (4.90)	28.91 (3.57)	28.94 (0.86)	27.75 (1.86)	27.64	26.67
Cognitive Z Scores	0.89 (2.62)	0.82 (1.89)	−0.02 (0.62)	0.01 (0.71)	0.84	0.75
Having *ABCA7* risk allele, (number)	1	2	0	1	1	1
Have Diabetes, (number)	0	1	0	1	0.0	0
Have Hypertension (number)	2	2	1	1	1	1
CSF Aβ42, pg/ml (SD)	133.34 (95.02)	139.81 (140.36)	200.93 (132.40)	164.27 (98.6)	201.11	134.69
CSF t-Tau, pg/ml (SD)	72.84 (49.63)	40.92 (20.79)	51.68 (30.96)	36.04 (11.63)	63.99	35.438
CSFp-Tau _181_, pg/ml (SD)	42.71 (20.98)	29.90 (15.60)	37.44 (16.29)	22.95 (8.46)	29.65	17.25
CSF t-Tau/Aβ42	0.79 (0.81)	0.26 (0.21)	0.85 (0.87)	0.15 (0.16)	0.46	0.22

### RsfMRI independent component analysis

We used a data driven approach (Independent Component Analysis; ICA) using the Group ICA of fMRI Toolbox v4.0b (GIFT) to identify large-scale brain networks [53, 54]. We first performed independent components analysis with model-order of 80 to empirically derive our regions of interest which enabled us to break the DMN into its various subregions, while still maintaining appropriate degrees of freedom. ICA is a data driven approach that allows for more adaptation to individual subject variability, which is essential in special populations, particularly those with atrophy as in our sample. The DMN and its subnetworks are relatively robust, and can easily be identified in a higher order ICA model [16, 55]. We chose an ICA approach as it can be more sensitive to sample characteristics, such as brain atrophy in older populations, than standard atlas based seed-regions while still accurately identifying regions of interest [56, 57].

**Fig. 1:**
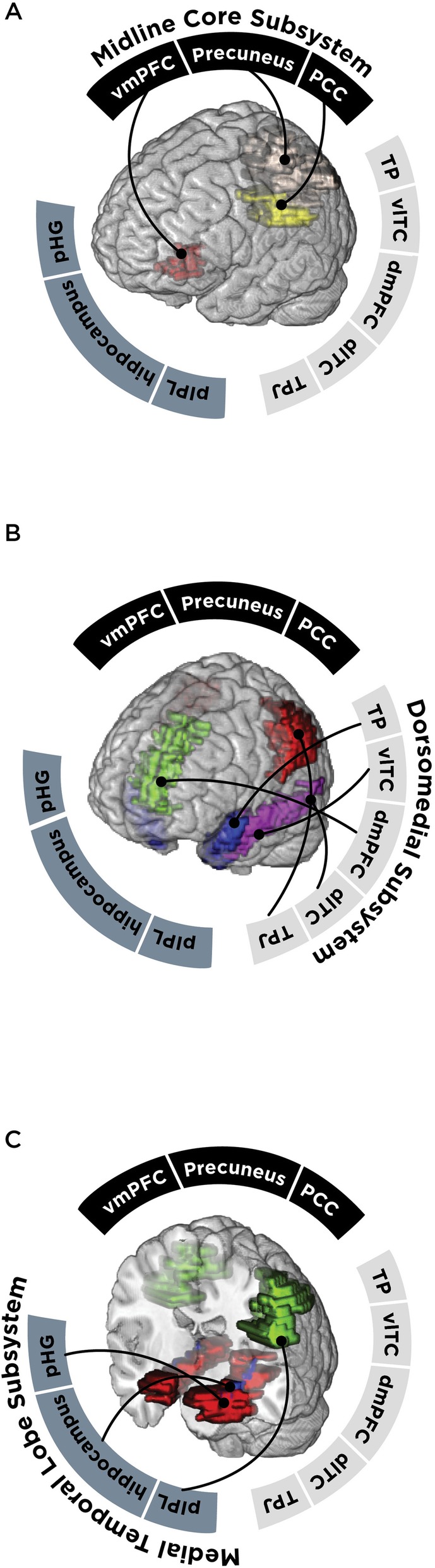
Empirically derived component maps of nodes according to each DMN subsystem. TP = temporal pole, vlTC = ventro-lateral temporal cortex, dmPFC = dorsomedial prefrontal cortex, dlTC = dorsolateral temporal cortex, TPJ = temporal parietal junction, pIPL = posterior inferior parietal lobule, pHG = parahippocampal gyrus, vmPFC = ventromedial prefrontal cortex, PCC = posterior cingulate cortex

To identify our regions of interest, we correlated all non-artifactual components [58] with templates of the DMN and chose components with the highest correlation values to the templates (0.80 cutoff threshold). Using the default mode network subdivisions and coordinates outlined by Andrews-Hanna [36], we identified 11 components that contained our regions of interest for the DMN subsystems. Components were manually confirmed using the xjview toolbox (http://www.alivelearn.net/xjview/) to ensure that they contained only our regions of interest. Regions included the temporal pole (TP), lateral temporal cortex (2 regions; ventrolateral (vlTC) and dorsolateral (dlTC), dorsomedial prefrontal cortex (dmPFC), and the temporal parietal junction (TPJ) which comprised the dorsomedial subsystem; the parahippocampal gyrus (pHG), hippocampus, and posterior inferior parietal lobule (pIPL) comprised the medial temporal lobe subsystem; finally the precuneus, posterior cingulate (PCC), and ventromedial prefrontal cortex (vmPFC) comprised the midline core subsystem (Fig. 1). We then calculated functional connectivity in GIFT by correlating the time courses of signal fluctuations between the chosen components, and obtained a correlation value for each region pair for a total of 55 measures of pairwise connectivity.

### Statistical analyses

Statistical analysis was performed in IBM SPSS 24.0 (Armonk, NY) and R version 3.3.3 [59]. MANCOVA was used to determine if race modifies DMN connectivity according to cognition. First, we analyzed baseline connectivity differences (only within controls). Measures of intra-network connectivity between the DMN nodes were the dependent variables; cognitive scores, race, gender, age, and MFWD were independent variables. Next, we analyzed data from all participants using the same model, but included a higher order interaction term (race X cognitive scores). Separate models to additionally account for effects of *APOE* ε4, *ABCA7* risk allele, hypertension, total WMH volume, cardiovascular risk score, and diabetes on DMN connectivity were also analyzed. For race-dependent connectivity changes, we accounted for multiple comparisons through the Benjamin-Hochberg method [60]. False discovery rate was limited to 10% given our sample size and the number of nominally significant interactions with race.

The same analysis was repeated according to Aβ42 levels in all subjects. Because there is significant overlap in t-Tau levels between NC and AD, we performed a third analysis according to t-Tau levels only in subjects with reduced Aβ42 levels (< 192 pg/mL) [61]consistent with cerebral amyloid deposition [62]. Compared to using uncorrected nominal *p* < 0.05 as a threshold, we reduced the number of race-dependent node pairs from 23 to ten (from six to four for cognition, from ten to two for CSF Aβ42, and from seven to four for CSF t-Tau). Because we observed an over-representation of race’s effect on inter-subsystem connectivity between nodes belonging to the midline core and dorsomedial (midline-dorsomedial) subsystems regardless of the measure used for AD (cognition, Aβ42, t-Tau), we used bootstrapping (see below) to test whether the midline-dorsomedial connectivity was preferentially modified by race in AD compared to intra-subsystem and other inter-subsystem node pairs. Finally, as confirmation, we used analysis of covariance (ANCOVA) to determine whether race influenced the mean midline-dorsomedial connectivity, midline-temporal connectivity, and dorsomedial-temporal connectivity adjusting for diagnosis, age, and gender. Mean subsystem connectivity [28] was calculated by averaging, for each individual, all pairwise inter-subsystem node pair connectivity between the two subsystems in question (15 pairs in midline-dorsomedial, 9 pairs in midline-temporal, and 15 pairs in dorsomedial-temporal).

### Bootstrapping

We developed a novel simulation-based approach to test whether there was empirical enrichment, or over-representation, for race modifying connectivity between midline core and dorsomedial subsystems. To determine the likelihood of a concentration of significant interaction terms occurring by chance alone, we first obtained *p*-values for all Race x Cognitive Z-score interaction term for all potential node pairings (*n* = 55; all subjects), and repeated the process for Aβ42 (n = 55; all subjects) and t-Tau (n = 55; only in subjects with Aβ42 < 192 pg/mL).

**Fig. 2:**
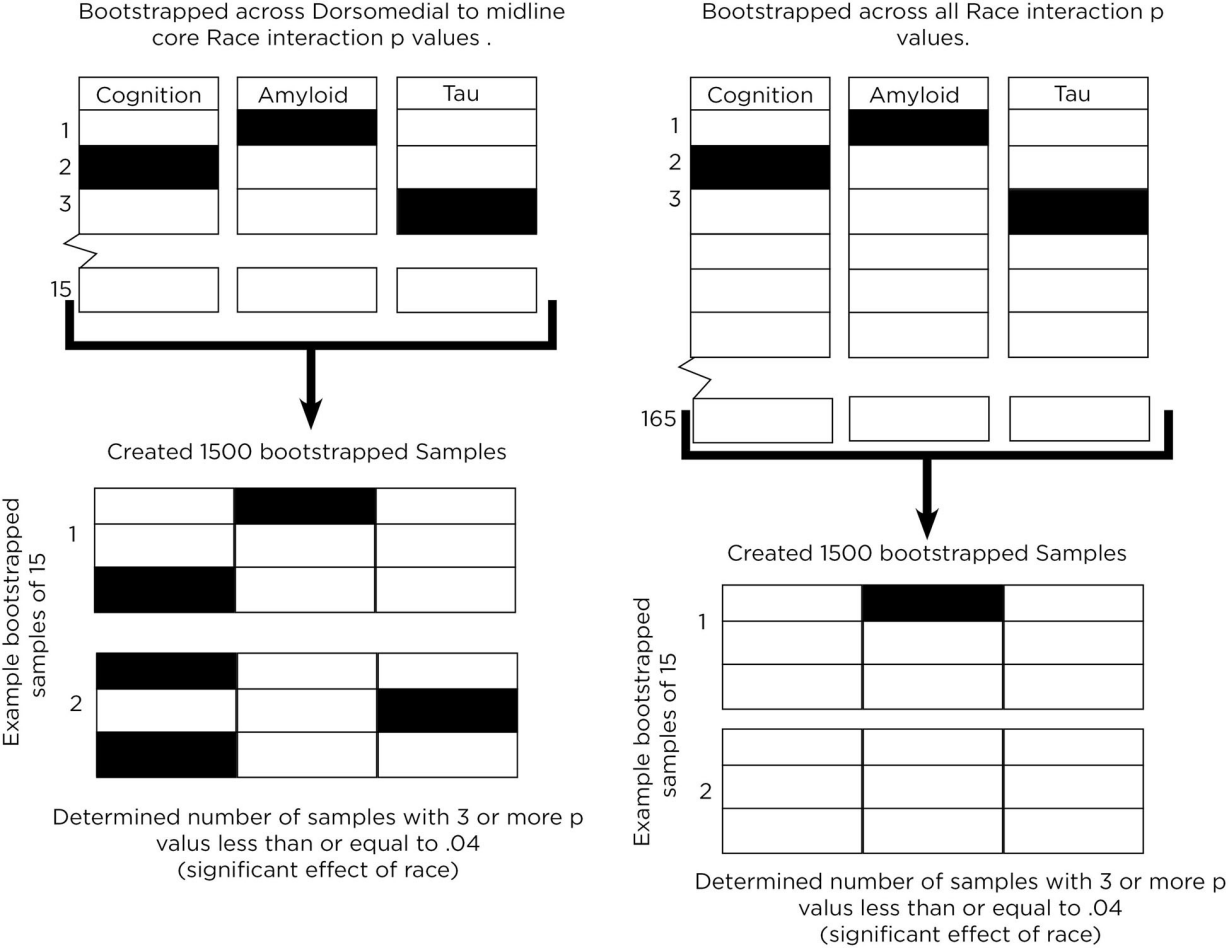
Illustrated workflow of the *p*-value bootstrapping analysis to confirm concentration of race’s effect on midline-dorsomedial connectivity. Filled boxes represent node-pair connectivity modified by race, and empty boxes represent node-pair connectivity not modified by race. In the first model, 15 *p*-values are selected randomly from any of the three midline-dorsomedial 3 × 5 matrices, and this was repeated to generate 1500 such sets of 15 *p*-values. In the second model, 15 p-values are selected randomly from all node pairs, and this was repeated 1500 times

As these AD features are inter-related, we pooled all 165 (55 × 3) *p*-values together, and used bootstrapping analysis (“boot” package in R [63], with replacement) to create 1500 simulated 3 × 5 (size of midline-dorsomedial matrix) matrices of *p*-values. The number of matrices (out of 1500) with three or more significant *p*-values is thus the probability of an observed concentration in any random 3 × 5 matrix of node pairs resulting from chance alone. At the same time, because this probability can be artificially reduced by a more stringent threshold at the matrix level (e.g., four or more significant *p*-values), we created a second set of 1500 simulated *p*-value matrices through the same bootstrapping process to represent the range of possible midline-dorsomedial p-values. Instead of drawing from all potential *p*-values, these 1500 matrices were then only sampled from *p*-values pooled from the 45 interaction *p*-values between midline-dorsomedial node pairs (*n* = 15 each for Race x Cognitive Z-score, Race x Aβ42, and Race x t-Tau) (Fig. 2). The probability of having three or more significant *p*-value in each matrix in this second bootstrap is then compared with the first using Chi-squared test. The null hypothesis for this test was that the number of samples that contained more than three significant *p* values would not differ between the midline-dorsomedial bootstrap and the chance-only bootstrap. We elected to use 1500 as the bootstrap size as it is well within the commonly recommended threshold [64], but still a tiny fraction of all possible combinations.

## Results

### Baseline connectivity differences

**Fig. 3:**
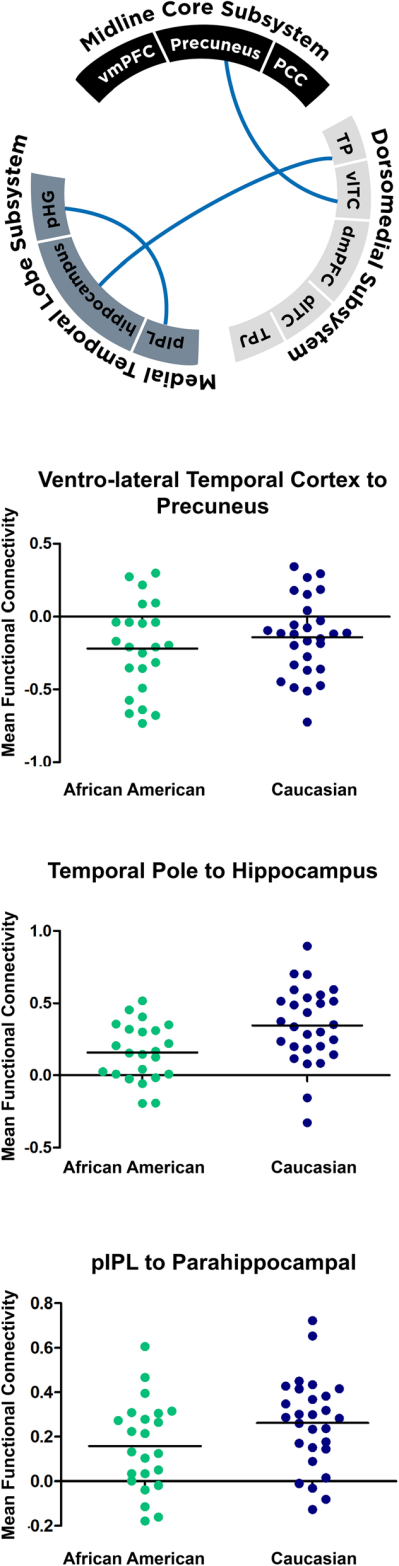
Baseline connectivity differences between older African Americans and Caucasians with NC (*p* < 0.04). Among nodes distributed along the three subsystems, three node pairs’ connectivity differed between the races. In all instances, African Americans (green) with NC had lower connectivity between these node pairs than Caucasians (blue; bars represent mean values). TP = temporal pole, vlTC = ventrolateral temporal cortex, dmPFC = dorsomedial prefrontal cortex, dlTC = dorsolateral temporal cortex, TPJ = temporal parietal junction, pIPL = posterior inferior parietal lobule, pHG = parahippocampal gyrus, vmPFC = ventro-medial prefrontal cortex, PCC = posterior cingulate cortex

We first compared baseline connectivity profiles between older African Americans and Caucasians with NC (*n* = 58, Fig. 3). Compared to Caucasians, African Americans had lower connectivity between the precuneus and the ventrolateral temporal cortex (by 0.31, 95% CI 0.16− 0.46, *p* = 0.01), the inferior parietal lobule and parrahippocampal gyrus (by − 0.15, 95% -0.28, − 0.03, *p* = 0.01), and the temporal pole and hippocampus (by 0.19, 95% CI 0.33− 0.04-0.33, *p* = 0.01; Table 3). There were otherwise no baseline connectivity differences in the remaining 52 inter-nodal connectivity values between the two racial groups.

**Table 3:** Baseline differences in functional connectivity between African Americans with NC and Caucasians with NC, adjusting for age, gender, and *APOE* ε4 allele

Connectivity	B (95% Confidence interval)	Unadjusted p	Storey’s q
Temporal Pole to Hippocampus	−0.19(− 0.33, − 0.04)	0.01	0.137
**Ventrolateral Temporal Cortex to Precuneus**	**−0.31(− 0.46, − 0.16)**	**0.01**	**< 0.001**
Inferior Parietal Lobule to Parahippocampal gyrus	− 0.15(− 0.28, − 0.03)	0.01	0.495

### Race-independent changes centered in the medial temporal lobe subsystem of DMN

**Fig. 4:**
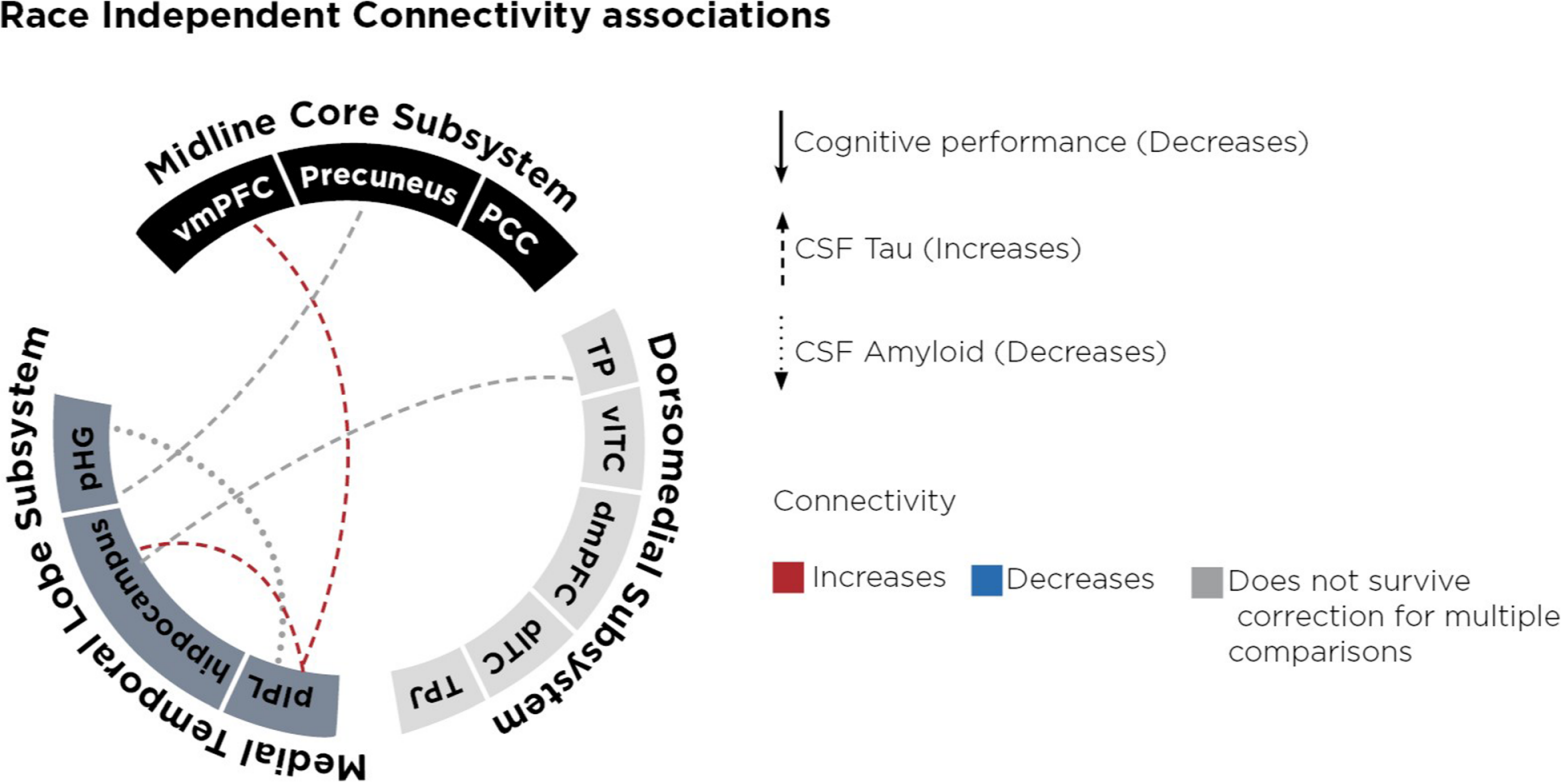
Race independent connectivity associations with biomarkers. Lines represent regions pairs for which connectivity was significantly related to the particular AD biomarker regardless of race (dashed line indicate CSF tau, solid line indicates cognition, and dotted line indicates CSF amyloid, red for positive relationship, blue for negative relationship, and grey for a relationship that did not survive correction for multiple comparisons). TP = temporal pole, vlTC = ventrolateral temporal cortex, dmPFC = dorsomedial prefrontal cortex, dlTC = dorsolateral temporal cortex, TPJ = temporal parietal junction, pIPL = posterior inferior parietal lobule, pHG = parahippocampal gyrus, vmPFC = ventromedial prefrontal cortex, PCC = posterior cingulate cortex

Because AD is characterized by reduced CSF Aβ42, increased CSF t-Tau, and cognitive impairment, we first analyzed the relationship between DMN connectivity, AD biomarkers (cognitive Z-score, Aβ42, t-Tau), and race to determine when race did not modify the relationship between AD biomarker and connectivity. In both African Americans and Caucasians, lower (more abnormal) Aβ42 levels correlated with decreased connectivity between the inferior parietal lobule and the parrahippocampal gyrus (*B* = −0.01, *t* (167) = − 2.14, *p* = 0.02). Because there is overlap in CSF t-Tau and p-Tau_181_ levels between controls and AD even though their levels are elevated at the group level, we restricted t-Tau-related analysis to those with Aβ42 levels consistent with AD (< 192 pg/mL). This also showed higher (more abnormal) t-Tau levels to correlate with decreased connectivity between multiple region pairs within the DMN, including hippocampus-temporal pole (*B* =0.04, *t* (167) = 1.58, *p* = 0.02) (Fig. 4). Connectivity correlated with cognitive impairment regardless of race appeared to occur between the medial temporal lobe and the midline core subsystems, and between the medial temporal lobe and the dorsomedial subsystems (Table 4, Fig. 4).

**Table 4:** Factors associated with AD biomarkers (unadjusted *p* ≤ 0.01) independent of race, adjusting for age, gender, mean framewise displacement, and *APOE* ε4 allele

Subsystems	Connectivity	Factor	B (95% Confidence Interval)	Unadjusted p	Storey’s q
MTL to MTL	Posterior IPL to Hippocampus	Race	0.019(− 0.29, − 0.05)	0.387	
		**t-Tau**	**0.002(0.00, 0.003)**	**< 0.001**	**< 0.001**
	Posterior IPL to Parahippocampal gyrus	Race	0.32(−0.14, .25)	0.710	
		Aβ42	0.02 (0.008, 0.40)	0.010	1.000
MTL to midline core	Posterior IPL to Ventromedial prefrontal cortex	Race	−0.05(−0.30, 0.20)	0.675	
		**t-Tau**	**0.002(0.00, 0.003)**	**0.001**	**0.004**
	Parahippocampal gyrus to Precuneus	Race	−0.10(−0.30, 0.23)	0.358	
		t-Tau	0.002 (0.00, 0.002)	0.008	0.147
MTL to dorsomedial	Temporal Pole to Hippocampus	Race	−0.10(−0.30, 0.10)	0.337	
		t-Tau	0.002(0.00, 0.003)	0.009	0.247

Unadjusted p-values which remain significant after Benjamini-Hochberg step-up correction for multiple comparisons are bolded. Storey’s q-values are also shown with FDR < 10%. *MTL* Medial Temporal Lobe, *IPL* inferior parietal lobule

### Race selectively modified the relationship between AD biomarkers and connectivity only between the MTL and Dorsomedial subsystem nodes

**Fig. 5:**
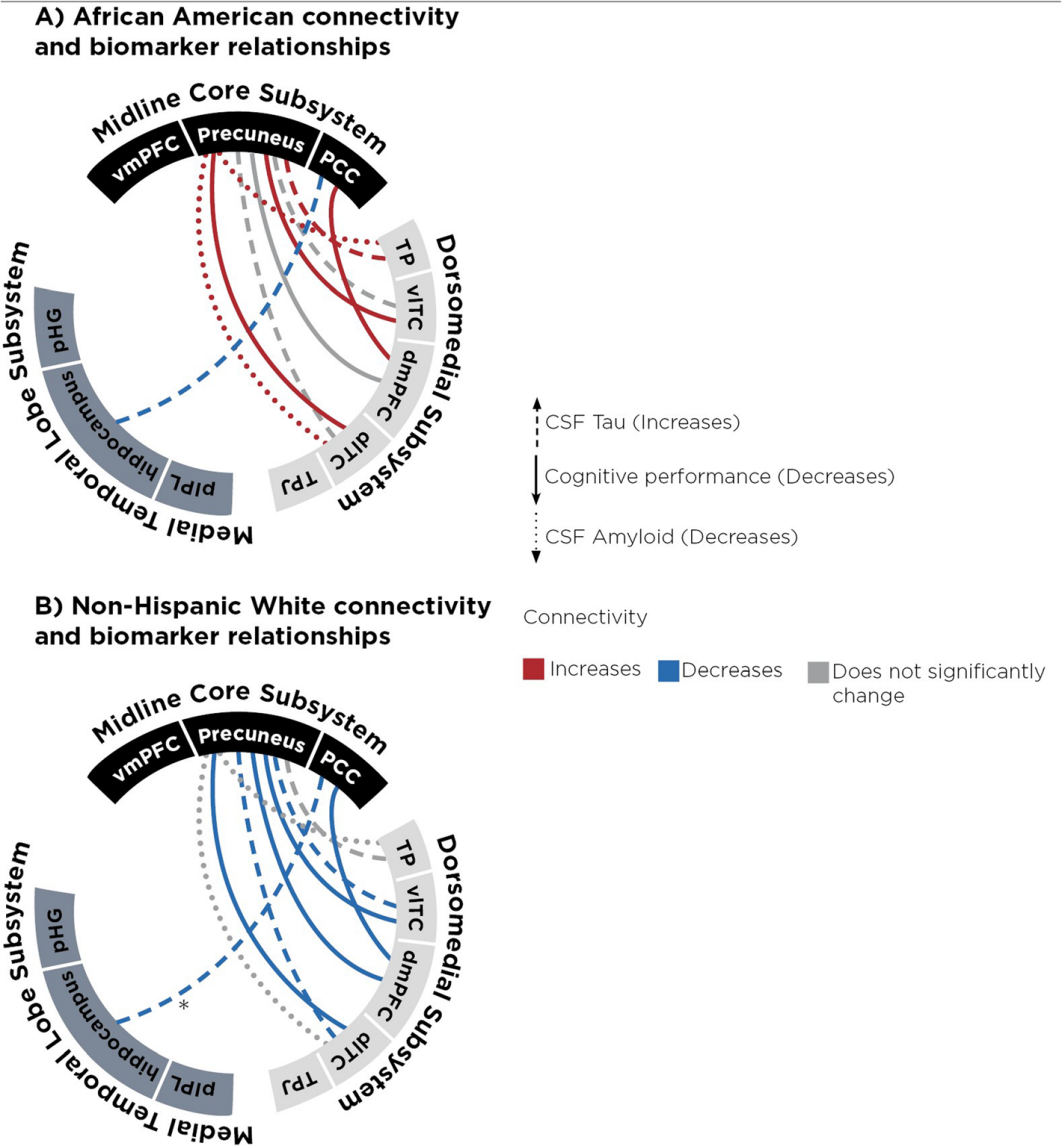
Connectivity and biomarker relationships in African Americans for which interaction term regression coefficient (race X biomarker) is significantly different from zero. Figure depicts regression relationship between connectivity and biomarkers in African Americans. Red line indicates connectivity increases as disease burden for that biomarker increases (see indication for each biomarker). Blue line indicates connectivity significantly decreases as disease burden for that biomarker increases (see indication for each biomarker). Gray outline indicates no significant relationship for African Americans between connectivity and biomarker. Dashed line indicate CSF tau, solid line indicates cognitive performance, and dotted line indicates CSF amyloid. * = indicates for that relationship, NHWs had significantly stronger (more negative) relationship than AAs. TP = temporal pole, vlTC = ventro-lateral temporal cortex, dmPFC = dorsomedial prefrontal cortex, dlTC = dorso-lateral temporal cortex, TPJ = temporal parietal junction, pIPL = posterior inferior parietal lobule, pHG = parrahippocampal gyrus, vmPFC = ventro-medial prefrontal cortex, PCC = posterior cingulate cortex

We next examined node pairs whose connectivity relationship with AD biomarkers was modified by race (Table 5, Fig. 5). In Caucasians, greater cognitive impairment was associated with decreased DMN connectivity between the precuneus and lateral temporal cortex, and between the precuneus and the temporal pole. However, the opposite is true in African Americans, with greater cognitive impairment associated with *increased* connectivity between these same regions. Similarly, lower (more abnormal) Aβ42 levels correlated with greater connectivity between the precuneus and both lateral temporal cortex and dorsomedial prefrontal cortex only in African Americans. Higher t-Tau levels (in those with Aβ42 levels < 192 pg/mL) also correlated with greater connectivity between the lateral temporal cortex and precuneus, and between the temporal pole and both vmPFC and precuneus, and between the hippocampus and PCC, again only in African Americans. Adjusting for risk genes (*ABCA7*, *APOE)* and other factors (hypertension, cardiovascular risk score, white matter hyperintensities, and diabetes) did not significantly influence connectivity values and race-associated differences persisted in connectivity relationship.

**Table 5:** Factors differentially associated with AD biomarkers according to race (unadjusted *p* ≤ 0.01), adjusting for age, gender, mean framewise displacement, and *APOE* ε4 allele

Subsystem	Connectivity Pair	Variable Name	B(95% Confidence Interval)	Unadjusted p	Storey’s q
Dorsomedial to midline core	Dorsomedial Prefrontal Cortex to Precuneus	Race	−0.02(−0.30, 0.25)	0.861	
		Cognitive impairment	0.04(−0.13, 0.04)	0.292	
		Race * Cognitive impairment	**−0.14(0.07, 0.25)**	**0.005**	**0.092**
	Ventrolateral Temporal Cortex to Precuneus	Race	−0.24(− 0.59, 0.11)	0.170	
		Cognitive impairment	−0.08(− 0.02, 0.19)	0.103	
		Race * Cognitive impairment	**0.18(0.02, 0.17)**	**0.001**	**0.055**
		Race	−0.05(−0.32, 0.21)	0.695	
		t-Tau	−0.003(− 0.02, − 0.002)	0.712	
		Race * t-Tau	**0.02(−0.03, − 0.0002)**	**0.001**	**0.055**
	Dorsomedial Prefrontal cortex to Posterior Cingulate Cortex	**Race**	**0.29(0.08, 0.50)**	**0.007**	
		Cognitive Impairment	0.05(−0.15, 0.03)	0.407	
		Race * Cognitive impairment	**0.18(0.02, 0.17)**	**0.007**	**0.096**
	Temporal Pole to Precuneus	Race	0.05(−0.18, −0.29)	0.667	
		Aβ42	0.0003(0.00, 0.01)	0.345	
		Race * Aβ42	**−0.001(−0.002, − 0.00001)**	**0.004**	**0.073**
		Race	−0.80(− 0.29, 0.13)	0.442	
		t-Tau	−0.002(− 0.01, − 0.001)	0.736	
		Race * t-Tau	**0.003(0.001, 0.006)**	**0.005**	**0.069**
	Dorsolateral Temporal Cortex to Precuneus	Race	−0.21(−0.12, 0.36)	0.112	
		Aβ42	0.0002(−0.0001, 0.01)	0.345	
		Race * Aβ42	**−0.002(− 0.003, − 0.00001)**	**0.002**	**0.055**
		Race	−0.21(− 0.43, 0.02)	0.070	
		t-Tau	−0.001(− 0.002, − 0.02)	0.066	
		Race * t-Tau	**0.004(0.001, 0.006)**	**0.003**	**0.083**
		**Race**	**−0.074(− 0.20,0.54)**	**0.004**	
		**Cognitive impairment**	**0.27(−0.04,0.09)**	**0.001**	
		Race * Cognitive impairment	**0.20(0.05, 0.25)**	**0.002**	**0.055**
MTL to midline core	Posterior Cingulate to Hippocampus	Race	−0.001(−0.22, 0.22)	0.900	
		t-Tau	−0.001(− 0.03, 0.002)	0.199	
		Race * t-Tau	**0.003(0.001, 0.052)**	**0.004**	**0.073**

Unadjusted p-values which remain significant after Benjamini-Hochberg step-up correction for multiple comparisons are bolded. Storey’s q-values are also shown with FDR < 10%. *MTL* Medial Temporal Lobe, *IPL* inferior parietal lobule

Visualizing race-independent (Fig. 4) and race-dependent (Fig. 5) DMN changes in AD, we observed a pattern of race-specific changes involving connectivity between two subsystems. Whereas race-independent connectivity occurred between each pair of subsystems, nine out of ten race-dependent connectivity changes were between the midline core and dorsomedial subsystems. For each subject, we calculated a mean connectivity value by averaging the all node-pair connectivity values between two subsystems. ANCOVA adjusting for diagnosis, age, and gender showed a main effect for race (F (2, 119) = 3.255, p = 0.074) for mean midline-dorsomedial connectivity, but not for mean midline-temporal connectivity (F (2,119) = 0.061, p = 0.8060) or mean dorsomedial-temporal connectivity (F (2,119) = 1.418, p = 0.236).

We further tested whether the midline-dorsomedial connectivity had an over-representation of node pairs whose connectivity was modified by race compared to the rest of DMN, we used bootstrapping (with replacement) to create 1500 simulated 3 × 5 matrices drawn from midline-dorsomedial node pairs and 1500 simulated matrices drawn from all node pairs. We found that drawing from the midline-dorsomedial matrices was more likely to result in identifying at least three significant race X AD biomarker effect than drawing from all node pairs: 791/1500 in the midline-dorsomedial sample vs. 192/1500 in the chance-only sample, *X*^*2*^ (2, *N* = 3000) = 487.53, *p* = 0.00001.

## Discussion

Consistent with previous work, we found AD to alter connectivity between the medial temporal lobe and dorsomedial subsystems, but we identified race-specific changes associated with these alterations [ 65, 66]. Importantly, we extend the effect of race on AD-related connectivity from the inter-nodal level to the inter-subsystem level through a novel analytical strategy. To the best of our knowledge, this is the first attempt to statistically identify enrichment of a factor’s effect on connectivity between two subsystems across multiple related measures (cognition, Aβ42, t-Tau). The implication of this inter-subsystem effect is not well understood. Other conditions previously observed to confer similar specificity on inter-subsystem connectivity include PTSD [36], depression [67], and schizophrenia [68]. Interestingly, some of these conditions show racial disparities (schizophrenia [69] and PTSD [70] are more common in African Americans than Caucasians). The inter-subsystem specificity may reflect shared vulnerability to neuropsychiatric disorders in African Americans, existence of disease subtypes, or divergent disease-associated pathways. We discuss these possibilities in the context of AD in African Americans below.

In contrast to a uniformly slow disease process in African Americans, it is possible that the different pathologic processes in AD may not proceed at the same pace in African Americans. In post-mortem studies of AD (involving primarily Caucasians), Aβ42-rich neuritic plaques are found early in the medial temporal as well as neocortical regions [24]. In contrast, tau-related changes appear in the medial temporal lobe before a stage-wise involvement of the frontal and then parietal cortical regions [24]. If we can interpret these observations as early co-localization of neuritic plaques and neurofibrillary tangles in the medial temporal lobe, the race-independent effect on inter-subsystem connectivity involving this region is in keeping with shared early AD changes by older African Americans and Caucasians when CSF Aβ42 alterations are detectable. The attenuation of midline-dorsomedial connectivity in African Americans could then be interpreted as early compensation when AD is mild, or as pathological hyper-connectivity [71, 72]. This would support the diminished cognitive reserve hypothesis in African Americans (potentially due to vascular disease [73]), and the prevailing longitudinal models that African Americans have slower decline in the presence of AD pathology [3, 74]. At the same time, the correspondence between ante-mortem DMN connectivity changes and post-mortem lesional mapping is known to be imperfect. For example, we found connectivity involving the posterior inferior parietal lobule (pIPL, a node in the medial temporal lobe subsystem) to be affected by AD independent of race. This may suggest pIPL to be a locus of early AD pathology, but neurofibrillary tangles do not appear in this region until later in AD [75].

For example, other than milder AD-related tau pathology, the selectivity of race for midline-dorsomedial connectivity could result from non-AD pathologies outside these two subsystems or neuro-protective changes along the tracts connecting two subsystems. Limited autopsy studies have shown African Americans more likely than Caucasians to have mixed AD and vascular lesions [76], and we previously showed in this cohort that African Americans experienced greater cognitive impact than Caucasians from the same degree of WMH [12]. In the current study, we did not find total WMH volume to be related to race and connectivity. However, the impact of regionally specific WMH has yet to be examined. The baseline differences in connectivity suggest existing differences in brain function separate from disease mechanisms that could be related to vascular disease, but the nature of these differences is not well understood, and the inclusion of vascular disease in our regression models did not alter our results. Although hypertension was more prevalent in our African American cohort and African Americans had elevated cardiovascular risk scores, including this in our analysiswhen we included this variable in our analyses, it did not explain the variability associated with race. Our identification of race-associated changes in midline-dorsomedial connectivity would support a search for WMH changes outside of these two subsystems. Alternatively, Caucasians may be more likely to have WMH between these two subsystems [77]. The vascular load in our cohort was mild to moderate, as it is not feasible, or ecologically valid to recruit older patients with minimal vascular disease. There are a variety of risk factors and contributing comorbidities for Alzheimer’s Disease. It is possible that the various risk factors associated with AD may be different across different ethnic groups, such that AAs may have an increased vascular component of AD, while exhibiting AD pathology sufficient to meet diagnostic thresholds. WMH and AD are not mutually exclusive, and many have stated that WMH are a core feature of AD [78] [79],, and a better predictor of disease burden in African Americans [80]. Future research will explore region-specific WMH between races and whether these differences relate to observed connectivity biomarker relationships.

It would be remissive to not explore social factors which may contribute to these biological disparities. The current work is the first to establish AD-related connectivity difference between races, and extends the neurobiological phenotype of AD in African Americans beyond a higher prevalence. How historical and current social inequalities may interact with genetic and environmental risks to give rise to these biological endpoints remains unknown. A variety of social disparities including income (amount vs. purchasing power), education (length vs. quality), and discrimination may additively or synergistically converge on the same biological endpoints. When analyzed separately, these factors may individually correlate with racial disparity but fail to capture the entire range of exposures facing different groups. For example, individuals who experience racial discrimination and perceive it as such are more likely to have higher blood pressure and increased psychological distress [81–83], which in turn are risk factors for AD [84]. Chronic stress also increases connectivity between the DMN and other networks at least in young adults [85], and may in part account for baseline and AD-related connectivity differences between the two racial groups. We did not include household income as a surrogate measure of lifelong socioeconomic status because the two measures poorly correlate in retired people, and the sample size limited our ability to interpret results when we introduced a measure such as the Area Deprivation Index [86]. A larger sample size will be necessary to test mediation effects between discrimination, stress, cardiovascular disease, and negative health outcomes, and cohort studies need to explore biologically meaningful methods to characterize individual and group-based experiences of injustice.

While we present the first biomarker-informed analysis of DMN inter-subsystem connectivity in African Americans, there are a number of limitations to our study. We tested two common AD risk genotypes as mediators for race-associated differences, but we did not perform extensive genomic association analysis because of sample size. While we observed multiple race-associated differences in DMN connectivity using ICA, we did not perform seed-based analysis of other large-scale brain networks (e.g., salience network). This cohort’s African American participants had similar years of education and socioeconomic status as their Caucasian counterparts, but other medical, psychiatric, or psychosocial differences could contribute to inter-subsystem connectivity differences. We did not identify a modifying effect of race on mean connectivity strength between the MTL and dorsomedial subsystem. Lastly, both racial groups include heterogeneous genetic backgrounds and in some cases mixed genetic heritage, so our results should be interpreted at the cohort level rather than the individual level. Nevertheless, we present additional evidence that AD is associated with systematic biomarker differences between older African Americans and Caucasians. Because CSF t-Tau-related findings similar to ours were replicated in a separate US cohort [87], independent replication of these DMN findings will further highlight the importance of diversity, inclusion, and disparities in on-going effort to elucidate mechanism-related biomarkers in AD.

## Conclusions

We previously identified that African Americans and Caucasians share the same AD-associated CSF alterations related to amyloid deposition, but different CSF tau biomarker levels regardless of AD status [14]. Here we extend our findings to show older African Americans and Caucasians have similar AD-associated subsystem connectivity changes involving the medial temporal subsystems. However, we also demonstrate race-specific patterns of connectivity between the midline core and dorsomedial subsystems, that are in-line with current studies that suggest divergent tau relationships between races. We thus propose adding DMN connectivity to the list of biomarkers with race-dependent alterations in AD. Similar to CSF, rsfMRI profiles for AD established in pre-dominantly Caucasian cohorts may under-diagnose the disease when applied directly to African Americans, and negatively impact the interpretation of clinical trial outcomes when rsfMRI is used as surrogate marker of AD. The current work further provides specific regions of interest for imaging-based and molecular investigation of disease mechanisms.

## Data Availability

The data that support the findings of this study are available from the corresponding author, upon reasonable request.

## Abbreviations

AA: African American
AD: Alzheimer’s Disease
CSF: Cerebrospinal fluid
MCI: Mild cognitive impairment
MRI: Magnetic resonance imaging
NC: Normal cognition
NHW: Non-Hispanic White
rsfMRI: Resting state functional MRI
WMH: White matter hyperintensities
